# News Items

**Published:** 1870-03

**Authors:** 


*News Items.*

BOTH the San Francisco medical journals are in full blast, notwithstanding the reported consolidation. Without meddling in the local controversy, we are sure that any move which should deprive the editorial fraternity of the Drs. Gibbons, would be one to be regretted by the profession. Their journal (*The Pacific*) has been and is a most valuable one.——The *Journal of the Gynæcological Society* chronicles a case of nymphomania, reported to the society with request for suggestions as to treatment. Coitus had been tried ineffectually, "but proved useless, from the fact that those employed could not exert themselves half enough for her satisfaction." The society report leaves the matter still somewhat obscure, viz.: "Dr. Perry remarked that in his own experience cases of nymphomania were very rare. He thought that, in twenty-five years experience, he had seen but two instances of the kind. Dr. Sullivan was inclined to think that the most successful treatment, in many instances, was to allow excessive intercourse." Are we to understand this as the conclusion of the society?——The same journal, through Dr. Storer, properly characterizes the false and filthy statements of Mrs. Dall's famous letter *anent* female physicians (indorsed in the *Independent* by William Lloyd Garrison).——The *Nashville Journal* is always spicy, readable and instructive. In the February number the editor comments upon the "New Medical Society" of Washington, organized to include "females and colored men," and in the course of his article gives some inside views of the workings of the American Medical Association in its various attempts to control the colleges. Alluding to the jeremiads of the *elevators*, he says: "It is consolatory to think, however, that the American system of manufacturing doctors, however defective and needing reformation, did, notwithstanding, turn out from its feeble machinery these very distinguished reformers, and thus, like Swallow Barn's Mill, on Apple-pie Creek, did some good before its water-power was dried up by the dog-days." —— The *Western Medical Journal*, heretofore published at Indianapolis, has been merged in a new paper, *The American Practitioner*, jointly edited by Dr. Yandell, of Louisville, and its former able and accomplished editor, Dr. Parvin. This is a powerful combination of talent, and promises a rich reward in success. —— The *Baltimore Medical Journal* is a new candidate for professional favor, edited by Drs. E. L. Howard and T. E. Latimer. It is a handsome monthly of sixty-four pages, at $4.00 a year. It promises "independence of all other interests, and will not be the organ of any corporation or clique," which insinuates that there are other interests, corporations or cliques, even in the monumental city — from which (in Chicago), good Lord, deliver us! —— The *Medical Bulletin*, of Baltimore, has taken on a new dress, and is edited with spirit and ability. —— The *Leavenworth Medical Journal*, also, is inspired by the new year, and has fitted itself with a new typographical suit. There is an independence and outspoken exposure of shams, pretences and pretenders, in this frontier exchange of ours, that commends it most fully to our affections. The February number is excellent in this regard, and we wish we had space for its indignant exposition of sundry "Home Physicians," *Medical Advisers*, etc. —— The Lexington papers chronicle the death of the widely known and distinguished surgeon and medical teacher, Professor Benj. W. Dudley, at the ripe age of eighty-five years. The *American Practitioner* promises a memoir of his life and writings in its next number. —— At the time of the death of Prof. Dunglison there had been issued of his works 160,000 volumes. —— The venerable Dr. Nathan R. Smith has resigned the chair of surgery he has so long filled in the University of Maryland. —— Prof. J. W. Freer, of Rush Medical College, has returned to Europe, where he will remain until the opening of the next annual session of the College. The course on physiology, which he has recently completed, is probably the first completely demonstrative one ever given in any college in this country. All his illustrations, experiments, vivisections, etc., have been most remarkably successful, evidencing an originality and facility scarcely surpassed either in this country or Europe. —— Dr. Hunt has recently performed the operation of transfusion successfully, a consumptive young lady in Wisconsin being the patient, and members of the family furnishing the blood. Of course, the kind of case was not desirable for the experiment. Details are promised for a subsequent number. —— We have been shown an improved eye-shade, which seems excellently well adapted to the purpose. The common shade is usually troublesome, both by direct compression, and by its interference with transpiration and evaporation, the breath, etc. The improvement consists essentially in adjusting the shade, with a spring, so that a space of half an inch, or more, is left between the upper border and the forehead. They may be found at the drug-store of Walker & Son, McVicker's Theatre building.

